# Light-Powered Late-Stage Alkylation of N‑Heteroaromatics in Flow

**DOI:** 10.1021/acscentsci.5c00926

**Published:** 2025-06-05

**Authors:** Shun Wang, Zhiwei Zuo

**Affiliations:** †​ State Key Laboratory of Organometallic Chemistry, 58309 Shanghai Institute of Organic Chemistry, University of Chinese Academy of Sciences, Chinese Academy of Science, Shanghai 200032, China

## Abstract

Continuous-flow photochemistry allows late-stage functionalization of N-heteroaromatics with gaseous alkane.

## Graphical abstract

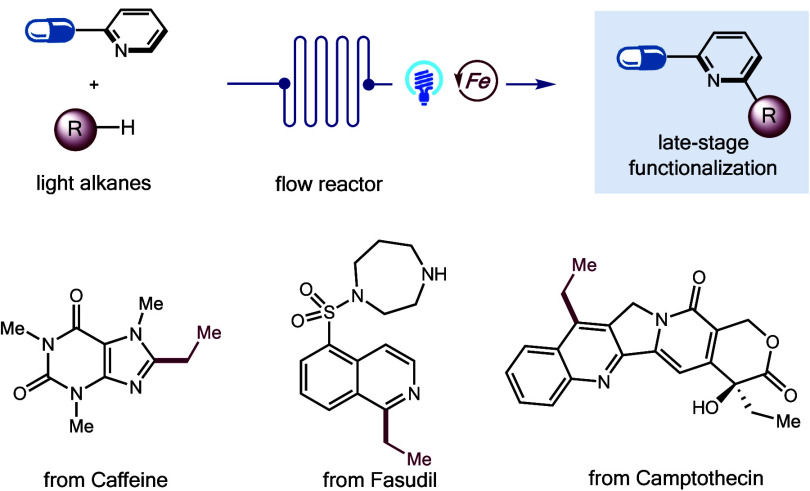

N-Heteroarenes are highly prevalent motifs in pharmaceuticals, agrochemicals, and small molecules of medicinal interest. Late-stage functionalization (LSF) of complex scaffolds containing N-heteroarenes is crucial for accelerating drug discovery efforts.^1^ The drug development process is complex, costly, and time-consuming. Direct C–H functionalization of approved drugs and bioactive compounds may result in compounds with improved properties, which can help accelerate the discovery of new drugs. Among available strategies, Minisci-type reactions have emerged as particularly effective for heteroarene functionalization, enabling nucleophilic alkyl radical addition to protonated heteroarenes under oxidative conditions. The past decade has witnessed the broad application of photoredox catalytic Minisci reactions, enabling the use of more abundant radical precursors to introduce diverse substituents onto heteroaromatics. In particular, the development of innovative photocatalytic HAT systems presents exciting opportunities for using simple and inexpensive inert feedstock gaseous alkanes as alkylation agents.^2^ Despite the tremendous progress, upscaling functionalization of poorly soluble and inert gaseous alkanes remains challenging, and the integration of photochemistry with flow technology holds potential for addressing this formidable challenge.^3,4^ While scattered reports from the author and others have demonstrated the benefits of operational simplicity and improving reaction efficacy in light alkane functionalization using photochemical flow systems, the application of these enabling techniques in the scalable LSF of complex molecules remains underexplored.^5^

In this issue of *ACS Central Science*, Noël and co-workers describe the photocatalytic late-stage alkylation of N-heteroarenes with gaseous alkanes in a continuous-flow setup.^6^ By strategically utilizing FeCl_3_ as a sustainable HAT catalyst and NFSI (N-fluoro-succinimide) as the sacrificial oxidant, the authors established an efficient photoinduced HAT system capable of activating gaseous C–H bonds in a custom-designed microtubing flow photoreactor. After systematic reaction screening in flow, the ethylation of quinoline with ethane gas under pressurized conditions was achieved with satisfactory efficiency under operationally simple conditions (Figure 1a).

**Figure 1:**
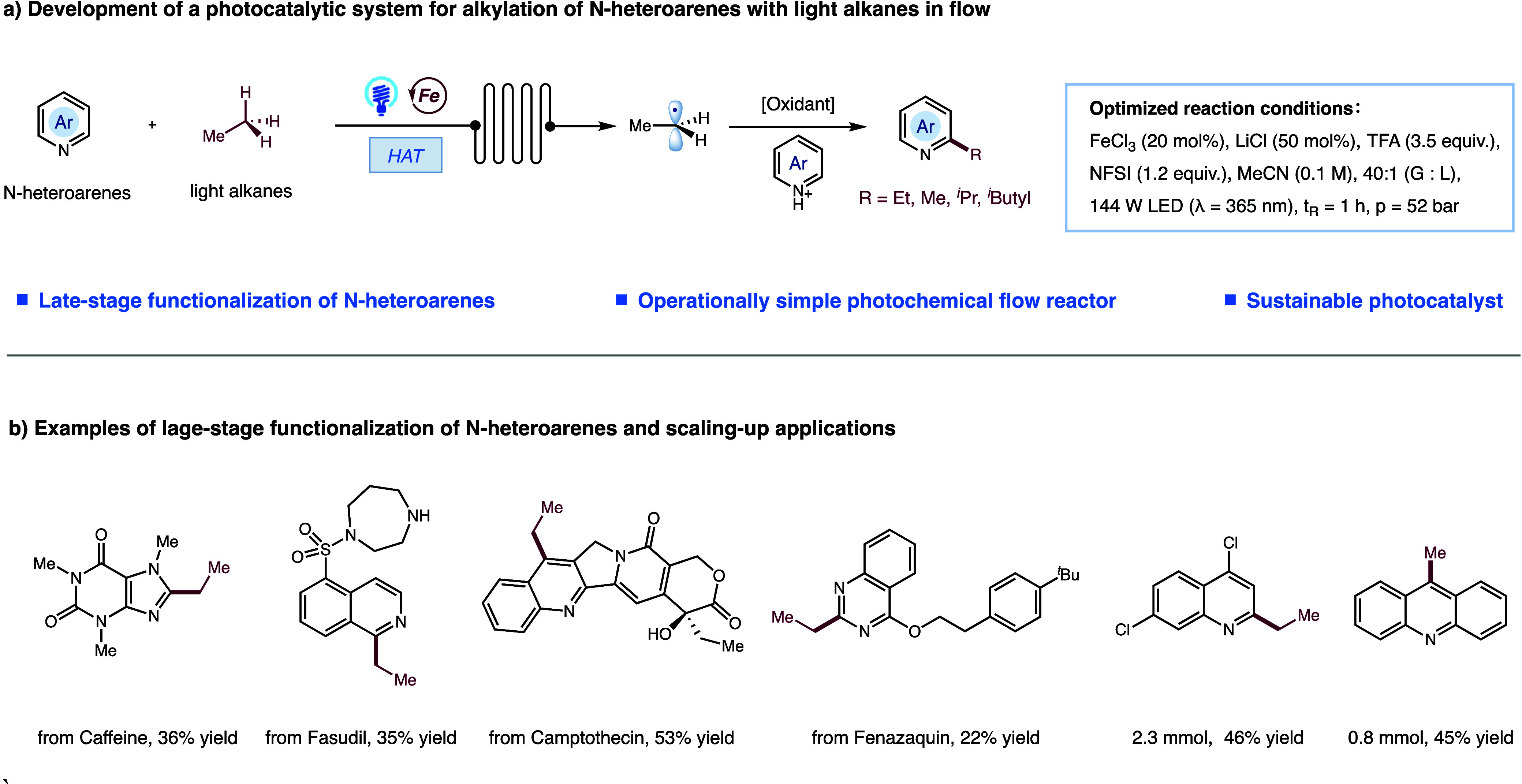
(a) Optimization of reaction conditions. (b) Representative examples of late-stage alkylation of N-heteroarenes and scaling-up applications.

The protocol exhibited excellent functional group tolerance, as demonstrated by its successful application to complex N-heteroarenes bearing sensitive functional groups, including halogen atoms, free amines, and alkenes.

Particularly noteworthy is the exceptional compatibility with relatively active C­(sp^3^)–H bonds in both the starting materials and products, compared to alkane C–H bonds, which benefits from the concentration effect.

While the application to marketed drug molecules afforded low to moderate yields, the fast reaction kinetics and operation simplicity make this protocol practical for drug modification (Figure 1b). The methodology was further validated by the alkylation of N-heteroarenes with other light alkanes (propane, butane, and methane). Notably, it demonstrated excellent scalability, which would considerably facilitate the process of drug development. By varying the light alkane feedstock, this protocol enabled rapid derivatization to access diverse alkylated heteroarenes. Moreover, the authors demonstrated the possibility of tuning the site selectivity in functionalization with ethane and butane by employing alkoxy radicals as the HAT species,^7^ which may allow precise late-stage alkylation of complex molecules.

Overall, this report shows that the integration of photocalysis with flow techniques features efficient light irradiation efficiency and enhanced gas–liquid mass transfer, which consequently allows the efficient and practical late-stage modification of N-heteroarenes using inert light alkanes as alkylating agents.

The simplicity and effectiveness of this photochemical flow approach, coupled with its compatibility with late-stage functionalization, position it as an enabling tool for pharmaceutical applications.
